# Facile synthesis of cyclopentenone B_1_- and L_1_-type phytoprostanes

**DOI:** 10.3389/fchem.2015.00041

**Published:** 2015-07-09

**Authors:** Alexandre Guy, Séamus Flanagan, Thierry Durand, Camille Oger, Jean-Marie Galano

**Affiliations:** Faculty of Pharmacy, Institut des Biomolécules Max Mousseron, UMR 5247 - Centre National de la Recherche Scientifique, University Montpellier École Nationale Supérieure de Chimie de MontpellierMontpellier, France

**Keywords:** phytoprostane, cross-metathesis, natural products, polyunsaturated fatty acids, oxygenated metabolites

## Abstract

Phytoprostanes (PhytoPs) represent non-enzymatic metabolites of α-linolenic acid (ALA), the essential omega-3 polyunsaturated fatty acid (PUFA) derived from plants. PhytoPs are present in the plant kingdom and represent endogenous mediators capable of protecting cells from oxidative stress damages in plants. Recently, it was found that such metabolites are present in cooking oil in high quantities, and also that B_1_-PhytoPs protect immature neurons from oxidant injury and promote differentiation of oligodendrocyte progenitors through PPAR-γ activation. We report a novel and facile synthesis of natural 2,3-substituted cyclopentenone PhytoPs, 16-B_1_-PhytoP, and 9-L_1_-PhytoP. Our strategy is based on reductive alkylation at the 2-position of 1,3-cyclopentanedione using a recent protocol developed by Ramachary et al. and on a cross-coupling metathesis to access conjugate dienone system. In conclusion, this strategy permitted access to B_1_- and L_1-PhytoPs_ in a relative short sequence process, and afford the possibility to easily develop analogs of PhytoPs.

## Introduction

Non-enzymatic production of biologically relevant compounds seems rather scarce in nature. One of the reasons could be the lack of researchers willing to accept the idea that auto-oxidative formation of metabolites could possess a primordial role in biology, and therefore to unravel their hidden properties. While this idea is only starting to grow in the field of oxidative stress and the production of non-enzymatically formed metabolites of polyunsaturated fatty acid (PUFA), it is still rather uncommon. In 1990, Morrow and Roberts discovered isoprostanes (IsoPs), compounds produced in human fluids and tissues by non-enzymatic free radical peroxidation of arachidonic acid (C20:4 n-3, AA) in phospholipid membranes (Morrow et al., 1990). These compounds later became the “gold standard” biomarker of lipid oxidative stress (Kadiiska et al., 2005), but also showed many relevant biological activities (Jahn et al., 2008; Galano et al., 2015). In 1998, Mueller and coworkers discovered that peroxidation of α-linolenic acid in plant (C18:3 n-3, ALA) lead to dinor-isoprostanes, later called Phytoprostanes (PhytoPs) (Parchmann and Mueller, 1998; Jahn et al., 2010). They also represent a standard of oxidative stress in plants (Durand et al., 2009), and are considered, like the jasmonate compounds, to be able to activate genes implicated in the detoxification response, and many other response signals to protect the plants (Durand et al., 2009). PhytoPs are also relevant to human, particularly to human diet, being present in vegetable oil (Karg et al., 2007; Collado-González et al., 2015a,b), and it was demonstrated that their level in humans can increase after high consumption of ALA (Barden et al., 2009). Recently, 2,3-substituted cyclopentenone PhytoPs such as 16-B_1_-PhytoPs showed protective effects on immature neurons from oxidant injury and on the differentiation of oligodendrocyte progenitors through PPAR-γ activation (Minghetti et al., 2014).

**Scheme 1:**
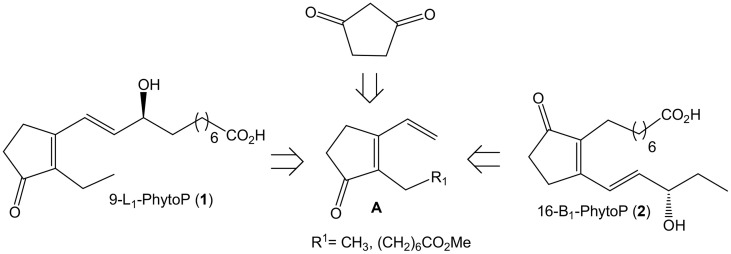
**Retrosynthetic analyses of 1 and 2 from 1,3-cyclopentanedione**.

A few syntheses of such 2,3-substituted cyclopentenone phytoprostanes are reported in the literature (El Fangour et al., 2005; Schmidt and Boland, 2007; Perlikowska and Mikołajczyk, 2009, 2011; Vázquez-Romero et al., 2009, 2013; Beretta et al., 2015), and our group developed the first ones in 2005, 9-L_1_-PhytoP and 16-B_1_-PhytoP (El Fangour et al., 2005). Later, Boland and coworkers developed a very elegant and rapid synthesis of such compounds and analogs based on a 1,3-cyclopentanedione functionalization and Heck or Sonogashira-type coupling reactions (Schmidt and Boland, 2007). We believed that a rapid and facile unexplored approach could also be carried out based on a cross-metathesis strategy between conjugated 3-vinyl-cyclopentenone derivatives such as **A** and enone or allyl unit partners to introduce the unsaturated side chain. Derivatives such as **A** could be obtained from 2-alkylation of 1,3-cyclopentanedione followed by introduction of the conjugated dienone unit (Scheme 1). Interestingly, large variation at the 2-position could be achieved using the elegant Ramachary protocol for reductive alkylation of 1,3-cyclopentanedione (Ramachary and Kishor, 2008). Here we described the rapid and facile syntheses of 2,3-substituted cyclopentenone PhytoPs, 16-B_1_-PhytoP and 9-L_1_-PhytoP based on the novel approach described above.

## Materials and methods

All reactions requiring anhydrous conditions were conducted in oven-dried glassware (120°C for 12 h minimum), syringes, and needles with magnetic stirring under nitrogen unless mentioned otherwise. Anhydrous THF and Et_2_O were obtained from the Innovative Technology PureSolv™ PS-400 solvent purification system. Other solvents and reagents were used as obtained from the supplier (Acros or Aldrich) unless otherwise noted. Lipase acrylic resin from *Candida antarctica* ≥5000 U/g, recombinant, expressed in *Aspergillus niger* was purchased from Sigma-Aldrich. Reactions were monitored by TLC using plates precoated with silica gel 60 (Merck). Reaction components were visualized by using a 254 nm UV lamp, treatment with acidic *p*-anisaldehyde stain followed by gentle heating. Organic layers were dried with anhydrous MgSO_4_ unless otherwise stated. Column chromatography was performed by using silica gel 40–63 μm. Optical rotations were measured with a Jasco P2000 digital polarimeter at 20°C. Concentrations *c* reported for the optical rotation data are given in g/100 mL. Infrared data are reported as wavenumbers (cm^−1^). ES-MS data were obtained by positive electrospray ionization methods. ^1^H NMR spectra were obtained at 300 or 500 MHz on Bruker spectrometers (AMX300 or Avance 500 MHz). The spectra were recorded in CDCl_3_ (internal reference at *δ* = 7.26 ppm) unless otherwise noted. The ^1^H NMR spectra are reported as follows: chemical shift in ppm [multiplicity, coupling constant(s) *J* in Hz, relative integral]. The multiplicities are defined as follows: br., broad; m, multiplet; AB, AB system; s, singlet; d, doublet; t, triplet; or combinations thereof. Selected ^13^C NMR spectra were recorded by using a *J*-modulated sequence, and the central peak of the CDCl_3_ triplet was used as the internal reference (*δ* = 77.16 ppm) and MeOD (fixed at *δ* = 49.0 ppm). The NMR spectra were assigned by homonuclear (^1^H–^1^H) and heteronuclear (^1^H–^13^C) correlation spectroscopy (COSY45, HSQC, HMBC). Infrared spectra were obtained using Perkin-Elmer Spectrum One Spectrometer. They were reported as wavenumber (cm^−1^) of significant peaks.

### 2-ethyl-3-methoxycyclopent-2-enone (3)

To a solution of 2-ethylcyclopentane-1,3-dione (1 g, 7.9 mmol, 1.0 eq) in 30 mL Et_2_O and 3 mL of MeOH, at 0°C, TMS-diazomethane solution was added (2M in Et_2_O, 6.9 mL, 9.9 mmol, 1.75 eq) over 1 min. After 10 min at 0°C and 2 h at room temperature, the reaction mixture was concentrated under reduced pressure. The crude was directly used in the next step without further purification. *R_f_* = 0.45 (95/5: EtOAc/MeOH);^1^H NMR (300 MHz, CDCl_3_): 3.89 (s, 3H), 2.61–2.58 (m, 2H), 2.40–2.36 (m, 2H), 2.10 (q, J = 7.5 Hz, 2H), 0.94 (t, J = 7.5 Hz, 3H); ^13^C NMR (75 MHz, CDCl_3_): δ = 204.7, 184.2, 122.1, 56.2, 33.4, 24.4, 14.4, 12.5.

### 2-ethyl-3-vinylcyclopent-2-enone (4)

To a crude solution of 2-ethyl-3-methoxycyclopent-2-enone **3** (theory ~ 7.9 mmol, 1.0 eq) in anhydrous THF (40 mL) at −78°C, a vinyl magnesium bromide solution (1M in THF, 11.9 mL, 11.9 mmol, 1.5 eq) was added dropwise. After 30 min at −78°C and 1 h at room temperature, the crude mixture was added to a solution of HCl (1M, 50 mL) and ice (80 g). The mixture was vigorously stirred 2 h and extracted with EtOAc (4 × 50 mL). The combined organic layers were washed with brine (3 × 50 mL), dried over anhydrous MgSO_4_, filtered and the solvents were removed under reduced pressure. The crude of the reaction was purified under silica gel chromatography (95/5 to 80/20: pentane/EtOAc). 663 mg of compound **4** was obtained (61% yield, 2 steps). *R_f_* = 0.63 (50/50: cyclohexane/EtOAc); ^1^H NMR (300 MHz, CDCl_3_): δ = 6.89 (dd, J = 10.7, 17.4 Hz, 1H), 5.70 (dd, J = 1.0, 17.4 Hz, 1H), 5.46 (dd, J = 1.0, 10.7 Hz, 1H); 2.62–2.59 (m, 2H); 2.39–2.36 (m, 2H), 2.25 (q, J = 7.6 Hz, 2H), 0.97 (J = 7.6 Hz, 3H); ^13^C NMR (75 MHz, CDCl_3_): δ = 209.5, 162.9, 143.0, 131.0, 120.7, 33.7, 24.9, 16.2, 13.3; HRMS (ESI^+^) calculated for C_9_H_13_O [M+H]^+^ 137.0966, found 137.0957.

### Methyl 9-hydroxyundec-10-enoate (5)

To a solution of methyl 10-undecenoate (1 g, 5 mmol, 1 eq) in 16 mL of CH_2_Cl_2_, SeO_2_ (1.11 g, 10 mmol, 2 eq) and *tert*-butyl hydroperoxide (5.5 M in decane, 6.5 mL, 35 mmol, 7 eq) were added. The mixture was heated under reflux overnight. After cooling, a solution of FeSO_4_ (11 g) and citric acid (3.5 g) in water (40 mL) was added slowly. The reaction mixture was extracted with Et_2_O (3 × 50 mL). The combined organic layers were washed with brine (3 × 50 mL), dried over anhydrous MgSO_4_, filtered and the solvents were removed without vacuum. The crude of the reaction was purified under silica gel chromatography (90/0 to 60/40: pentane/Et_2_O) to give 659 mg of **5** (61% yield). *R_f_* = 0.4 (70/30: cyclohexane/EtOAc); ^1^H NMR (300 MHz, CDCl_3_): δ = 5.83 (ddd, J = 6.3, 10.4, 17.2 Hz, 1H), 5.18 (dt, J = 1.3, 17.2 Hz, 1H), 5.06 (dt, J = 1.2, 10.4 Hz, 1H), 4.05 (q, J = 6.3 Hz, 1H), 3.63 (s, 3H), 2.26 (t, J = 7.5 Hz, 2 H), 1.65–1.46 (m, 5H), 1.32–1.21 (m, 8H); ^13^C NMR (75 MHz, CDCl_3_): δ = 174.2, 141.3, 114.4, 73.1, 51.3, 36.9, 34.0, 29.2, 29.0, 28.9, 25.1, 24.8; HRMS (ESI^+^) calculated for C_12_H_23_O_3_ [M+H]^+^ 215.1647, found 215.1658.

### Methyl 9-oxoundec-10-enoate (6)

To a solution of oxalyl chloride (304 μL, 3.5 mmol, 1.5 eq) in 15 mL of CH_2_Cl_2_ at −78°C, DMSO (497 μL, 7 mmol, 3 eq) was added dropwise. The mixture was stirred 10 min and a solution of **5** (500 mg, 2.33 mmol, 1 eq) in 5 mL of CH_2_Cl_2_was added. The crude was stirred 20 min and Et_3_N (1.95 mL, 14 mmol, 6 eq) was added. After stirring 2 h at room temperature, water (50 mL) was added. The layers were separated. The aqueous one was extracted with CH_2_Cl_2_ (2 × 25 mL) and the combined organic layers were washed with water (25 mL), saturated aqueous solution of NaHCO_3_ (25 ml), brine (20 mL) and dried over anhydrous MgSO_4_. The solvents were removed without vacuum. The crude of the reaction was purified under silica gel chromatography (95/5 to 80/20: pentane/Et_2_O) to give 389 mg of **6** (79% yield). *R_f_* = 0.6 (50/50: cyclohexane/EtOAc); ^1^H NMR (300 MHz, CDCl_3_): δ = 6.32 (dd, J = 10.3, 17.6 Hz, 1H), 6.17 (dd, J 1.2, 17.6 Hz, 1H), 5.77 (dd, J = 1.2, 10.3 Hz, 1H), 3.63 (s, 3H), 2.54 (t, J = 7.4 Hz, 2H), 2.26 (t, J = 7.5 Hz, 2H), 1.64–1.56 (m, 4H), 1.36–1.24 (m, 6H); ^13^C NMR (75 MHz, CDCl_3_): δ = 200.9, 174.1, 136.5, 127.3, 51.3, 39.5, 33.9, 28.9 (2 C), 28.8, 24.8, 28.8; HRMS (ESI^+^) calculated for C_12_H_21_O_3_ [M+H]^+^ 213.1491, found 213.1471.

### Enzymatic resolution of methyl 9-hydroxyundec-10-enoate (5)

To a solution of methyl 9-hydroxyundec-10-enoate **5** (1.2 g, 5.6 mmol, 1 eq) in hexane (5.2 mL) *Candida antarctica lipase* B (240 mg, 20% w/w) and vinyl acetate (5.2 mL, 56 mmol, 10 eq) were added. The reaction mixture was stirred overnight and filtered. The solvents were removed under reduced pressure. The crude of the reaction was purified under silica gel chromatography (90/10 to 60/40: pentane/Et_2_O) to give 657 mg of acetylated product (*R*)-**5-Ac** (93%ee, 46% yield) and 545 mg of free hydroxylated *(S)*-**5** (45% yield, 99% ee, [α]^20^_*D*_ = −5.3 (c 10, CHCl3).

(*R*)-**5-Ac** was deacetylated in the presence of K_2_CO_3_ (1.06 g, 7.7 mmol, 3 eq) in MeOH (50 mL). The mixture was stirred 5 h and brine (150 ml) was added. The crude was extracted with pentane/Et_2_O 1/1 (3 × 150 mL). The organic layers were washed with brine (3 × 100 mL), dried over anhydrous MgSO_4_ and evaporated slowly under controlled vacuum to obtain 505 mg of (*R*)-**5** (92% yield). (*R*)-**5** was enzymatically resolved one more time with CALB, deacetylated with K_2_CO_3_ and analyzed to give 333 mg of (*R*)-**5** (83% yield, ee = 99%).

Esterification of (*R*)-**5** and *(S)*-**5** with 2,4-dinitrobenzyl chloride in pyridine gave the corresponding esters, which were analyzed by chiral HPLC (Chiralcel OD®, 95/5: Hexane/isopropanol, 0.7 ml/min, λ = 254 nm) to give ee = 99% for *(S)*-**5-ester** (tr = 18.1 min) and ee = 93% for (*R*)-**5-ester** (tr = 23.6 min).

### Determination of stereochemistry of 5

One aliquot of *(S)*-**5** was esterified with (*S*)-acetyl phenyl acetic acid and (*R*)-acetyl phenyl acetic acid in presence of EDCI, DMAP in CH_2_Cl_2_. In the same way, *(R)*-**5** was esterified. With the four diastereoisomers in hand, the stereochemistry of *(S)*-**5** and *(R)*-**5** was determined as previously described (Chataigner et al., 1998).

### 9-oxo-9-L_1_-PhytoP (9)

To a mixture of **4** (200 mg, 1.47 mmol, 1 eq) and **6** (935 mg, 4.4 mmol, 3 eq) in carefully degassed CH_2_Cl_2_ (30 mL) Hoveyda Grubbs 2nd generation catalyst (69 mg, 0.11 mmol, 0.025 eq) was added. The mixture was heated 5 h under reflux. The crude was evaporated without heating with celite® and rapidly purified under silica gel chromatography (100/0 to 80/20: CH_2_Cl_2_/Et_2_O). The 9-oxo-9-L_1_-PhytoP methyl ester **8** was slightly degraded by silica gel and contaminated by dimer of **4** and **6**. The mixture was used directly in the next step.

To a solution of previously recovered 9-oxo-9-L_1_-PhytoP methyl ester **8** (and dimers) in MeCN (20 mL) and phosphate buffer (20 mL, pH = 7) CALB (20 mg, 2% w/w) was added. The reaction mixture was stirred overnight. The crude was extracted with CH_2_Cl_2_ (3 × 50 mL). The organic layers were washed with brine (2 × 50 mL) and dried over anhydrous MgSO_4_. The solvents were removed under vacuum. The crude of the reaction was purified under silica gel chromatography (100/0 to 98/2: CH_2_Cl_2_/MeOH) to give 260 mg of 9-oxo-9-L_1_-PhytoP **9** (58% yield, 2 steps) with 5% of *cis* isomer. *R_f_* = 0.25 (95/5: CH_2_Cl_2_/MeOH); ^1^H NMR (300 MHz, CDCl_3_): δ = 7.59 (d, J = 15.9 Hz, 1H), 6.51 (d, J = 15.9 Hz, 1H), 2.65–2.58 (m, 4H), 2.45–2.42 (m, 2H), 2.38–2.27 (m, 4H), 1.63–1.56 (m, 4 H), 1.36–1.23 (m, 6H), 1.00 (t, J = 7.5 Hz, 3H); ^13^C NMR (75 MHz, CDCl_3_): δ = 209.0, 200.1, 179.3, 159.7, 149.1, 133.9, 130.5, 41.4, 33.9, 33.8, 28.9, 28.8, 28.7, 25.3, 24.5, 23.8, 16.7, 13.5; HRMS (ESI^+^) calculated for C_18_H_27_O_4_ [M+H]^+^ 307.1909, found 307.1897.

### 9-L_1_-PhytoP methyl ester (*(S)*-7)

To a mixture of **4** (109 mg, 0.8 mmol, 1 eq) and *(S)*-**5** (214 mg, 1 mmol, 1.25 eq) in carefully degassed CH_2_Cl_2_ (15 mL) Hoveyda Grubbs 2nd generation catalyst (12.5 mg, 0.02 mmol, 0.025 eq) was added. The mixture was stirred for 2 days. A new crop of catalyst (12.5 mg) was added and the reaction was stirred for 3 more days. To the reaction mixture celite® was added followed by evaporation of the solvents without heating, then rapid purification under silica gel chromatography (95/5 to 50/50: CH_2_Cl_2_/EtOAc). 103 mg of 9-L_1_-PhytoP methyl ester *(S)*-**7** was obtained (40% yield). *R_f_* = 0.24 (80/20: CH_2_Cl_2_/EtOAc); ^1^H NMR (300 MHz, CDCl_3_): δ = 6.74 (d, J = 15.7 Hz, 1H), 6.19 (dd, J = 5.9, 15.7 Hz, 1H), 4.27–4.23 (m, 1H), 3.58 (s, 3H), 2.58–2.55 (m, 2H), 2.46 (sl, 1H), 2.35–2.31 (m, 2H), 2.25–2.18 (m, 4H), 1.57–1.51 (m, 4H), 1.36–1.20 (m, 8H), 0.93 (t, J = 7.5 Hz, 3H); ^13^C NMR (75 MHz, CDCl_3_): δ = 209.5, 174.2, 162.9, 142.4, 140.5, 123.4, 72.1, 51.3, 37.1, 33.9, 33.7, 29.2, 29.0, 28.9, 25.5, 25.2, 24.7, 16.2, 13.3; HRMS (ESI^+^) calculated for C_19_H_31_O_4_ [M+H]^+^ 323.2222, found 323.2207.

### *Ent*-9-L_1_-PhytoP methyl ester (*(R)*-7)

Similar to the above, e*nt*-9-L_1_-PhytoP methyl ester *(R)*-**7** was obtained from **4** and *(R)*-**5** in similar yield (108 mg, 42% yield). *R_f_* = 0.24 (80/20: CH_2_Cl_2_/EtOAc); ^1^H NMR (300 MHz, CDCl_3_): δ = 6.76 (d, J = 15.7 Hz, 1H), 6.20 (dd, J = 5.9, 15.7 Hz, 1H), 4.29–4.25 (m, 1H), 3.61 (s, 3H), 2.60–2.57 (m, 2H), 2.38–2.35 (m, 2H), 2.28–2.21 (m, 4H), 2.12 (sl, 1H), 1.59–1.53 (m, 4H), 1.36–1.23 (m, 8H), 0.97 (t, J = 7.5 Hz, 3H); ^13^C NMR (75 MHz, CDCl_3_): δ = 209.4, 174.2, 162.7, 142.6, 140.2, 123.6, 72.2, 51.3, 37.1, 33.9, 33.8, 29.2, 29.0, 28.9, 25.5, 25.2, 24.7, 16.2, 13.3.

### 9-L_1_-PhytoP (1)

To a solution of 9-L_1_-PhytoP methyl ester *(S)*-**7** (80 mg, 0.248 mmol, 1 eq) in MeCN (4.5 mL) and phosphate buffer (4.5 mL, pH = 7) CALB (1.6 mg, 2% w/w) was added. The reaction mixture was stirred for 1 day. The crude was extracted with EtOAc (3 × 10 mL). The organic layers were washed with brine (2 × 10 mL) and dried over anhydrous MgSO_4_. The solvents were removed under vacuum. The crude of the reaction was purified under silica gel chromatography (50/50 to 25/75: CH_2_Cl_2_/EtOAc) to give 63.4 mg of 9-L_1_-PhytoP **1** (83% yield). *R_f_* = 0.20 (95/5: CH_2_Cl_2_/MeOH); ^1^H NMR (500 MHz, CDCl_3_): δ = 6.81 (d, J = 15.7 Hz, 1H), 6.25 (dd, J = 6.0, 15.7 Hz, 1H), 4.34 (q, J = 6 Hz, 1H), 2.66–2.2.64 (m, 2H), 2.45–2.43 (m, 2H), 2.35 (t, J = 7.5 Hz, 2H), 2.31 (q, J = 7.5 Hz, 2H), 1.65–1.60 (m, 4H), 1.36–1.33 (m, 8H), 1.01 (t, J = 7.5 Hz, 3H); ^13^C NMR (125 MHz, CDCl_3_): δ = 211.1, 180.3, 164.2, 144.2, 141.4, 125.2, 73.8, 38.5, 35.2(2C), 30.6, 30.5, 30.3, 27.0, 26.6, 26.0, 17.7, 14.9; HRMS (ESI^+^) calculated for C_18_H_29_O_4_ [M+H]^+^ 309.2066, found 309.2070; [α]^20^_*D*_ = −19.3 (c 10, CHCl_3_).

### *Ent*-9-L_1_-PhytoP (*ent*-1)

Similar to the above, the *ent*-9-L_1_-PhytoP (*ent*-**1**) was obtained from *(R)*-**7** (69.2 mg, 85% yield). *R_f_* = 0.20 (95/5: CH_2_Cl_2_/MeOH); ^1^H NMR (500 MHz, CDCl_3_): δ = 6.81 (d, J = 15.7 Hz, 1H), 6.25 (dd, J = 6.0, 15.7 Hz, 1H), 4.34 (q, J = 6 Hz, 1H), 2.65–2.2.64 (m, 2H), 2.45–2.42 (m, 2H), 2.35 (t, J = 7.5 Hz, 2H), 2.31 (q, J = 7.5 Hz, 2H), 1.65–1.60 (m, 4H), 1.36–1.33 (m, 8H), 1.01 (t, J = 7.5 Hz, 3H); ^13^C NMR (125 MHz, CDCl_3_): δ = 211.1, 180.5, 164.3, 144.1, 141.5, 125.2, 73.8, 38.5, 35.3, 35.2, 30.6, 30.5, 30.3, 27.0, 26.6, 26.0, 17.7, 14.9; HRMS (ESI^+^) calculated for C_18_H_29_O_4_ [M+H]^+^ 309.2066, found 309.2043; [α]^20^_*D*_ = +19.1 (c 10, CHCl_3_).

### Methyl 8-oxooctanoate (10)

In a solution of cyclooctene (1.95 mL, 15 mmol, 1 eq) and Na_2_CO_3_ (400 mg, 3.75 mmol, 0.25 eq) in CH_2_Cl_2_/MeOH (45 mL/5 mL), at −78°C, a flow of generated ozone/air was bubbled 3 h, until a blue color appeared. After elimination of ozone excess by bubbling nitrogen, the reaction mixture was warmed to room temperature, then the solution was filtered to remove excess Na_2_CO_3_. The solvents were evaporated under reduced pressure. CH_2_Cl_2_(40 mL) was added to the crude reaction mixture, then Et_3_N (3.13 mL, 22.5 mmol, 1.5 eq) and Ac_2_O (3.9 mL, 41.5 mmol, 2.75 eq) were added successively dropwise. The mixture was stirred overnight at rt, washed with 0.1N HCl solution (2 × 30 mL), 1N NaOH solution (2 × 30 mL), water (30 mL), and brine (2 × 30 mL). The organic layer was dried over anhydrous MgSO_4_ and evaporated under reduced pressure. The crude was purified under silica gel chromatography (95/5 to 90/10: cyclohexane/EtOAc) to give 1.78 g of **10** (69% yield). *R_f_* = 0.33 (80/20: cyclohexane/EtOAc); ^1^H NMR (300 MHz, CDCl_3_): δ = 9.68 (t, J = 1.8 Hz, 1H), 3.58 (s, 3H), 2.35 (dt, J = 1.8, 7.3 Hz, 2H), 2.23 (t, J = 7.5 Hz, 2H), 1.58–1.51 (m, 4H), 1.29–1.24 (m, 4H); ^13^C NMR (75 MHz, CDCl_3_): δ = 202.5, 174.0, 51.3, 43.6, 33.8, 28.8, 28.6, 24.5, 21.7; HRMS (ESI^+^) calculated for C_9_H_17_O_3_ [M+H]^+^ 173.1178, found 173.1184.

### Methyl 8-(2-methoxy-5-oxocyclopent-1-enyl)octanoate (11)

To a solution of 1,3-cyclopentanedione (456 mg, 4.65 mmol, 1 eq) in CH_2_Cl_2_ (15 mL) at room temperature methyl 8-oxooctanoate **10** (1.2 g, 6.96 mmol, 1.5 eq), Hantzsch ester (1.3 g, 5.1 mmol, 1.1 eq), and L-proline (27 mg, 0.23 mmol, 0.05 eq) were added successively. The reaction mixture was stirred 4 h and the solvent was removed by evaporation under reduced pressure. A mixture of solvents Et_2_O/MeOH 9/1 (15 mL) was added to the crude and a solution of (trimethylsilyl)diazomethane (2M in Et_2_O, 4.64 mL, 9.28 mmol, 2 eq) was added dropwise. The mixture was stirred 30 min and solvents were evaporated under reduced pressure. The crude was purified under silica gel chromatography (75/25 to 50/50: CH_2_Cl_2_/EtOAc) to give 1.15 g of **11** (91% yield). *R_f_* = 0.44 (95/5: EtOAc/MeOH); ^1^H NMR (300 MHz, CDCl_3_): 3.87 (s, 3H), 3.59 (s, 3H), 2.61–2.58 (m, 2H), 2.38–2.35 (m, 2H), 2.22 (t, J = 7.4 Hz, 2H), 2.04 (t, J = 7.2 Hz, 2 H), 1.57–1.50 (m, 2H), 1.35–1.19 (m, 8H);^13^C NMR (75 MHz, CDCl_3_): δ = 204.8, 184.5, 174.2, 120.8, 56.2, 51.3, 34.0, 33.4, 29.2, 28.9, 28.8, 27.7, 24.8, 24.3, 21.1; HRMS (ESI^+^) calculated for C_15_H_25_O_4_ [M+H]^+^ 269.1753, found 269.1745.

### 8-(2-methoxy-5-oxocyclopent-1-enyl)octanoic acid (12)

To a solution of methyl ester **11** (1.4 g, 5.22 mmol, 1 eq) in MeCN (50 mL) and phosphate buffer (50 mL, pH = 7) CALB (28 mg, 2% w/w) was added. The reaction mixture was stirred for 2 days. After adding NaCl powder, the crude was extracted with EtOAc (3 × 50 mL). The organic layers were washed with brine (2 × 25 mL), and dried over anhydrous MgSO_4_. The solvents were removed under vacuum. The crude of the reaction mixture (**12**) (1.342 g) was used directly in the next step.^1^H NMR (300 MHz, CDCl_3_): 3.90 (s, 3H), 2.63–2.60 (m, 2H), 2.43–2.40 (m, 2H), 2.29 (t, J = 7.5 Hz, 2H), 2.09–2.00 (m, 2 H), 1.60–1.56 (m, 2H), 1.36–1.20 (m, 8H);^13^C NMR (75 MHz, CDCl_3_): δ = 205.3, 184.9, 179.0, 120.9, 56.3, 33.9, 33.3, 29.2, 28.9, 28.8, 27.7, 24.6, 24.4, 21.0.

### Methyl 8-(5-oxo-2-vinylcyclopent-1-enyl)octanoate (13)

To a solution of **12** (theory: 5.22 mmol, 1 eq), in anhydrous THF (55 mL), at −78°C, a vinyl magnesium bromide solution (1M in THF, 26.4 mL, 26.4 mmol, 5 eq) was added. After 30 min at −78°C and 2 h at room temperature, the crude reaction mixture was added to a solution of HCl (1 M, 150 mL) and ice (150 g). The mixture was vigorously stirred 2 h and extracted with EtOAc (4 × 100 mL). The combined organic layers were washed with brine (3 × 50 mL), dried over anhydrous MgSO_4_, filtered and the solvents were removed under reduced pressure. A mixture of solvents Et_2_O/MeOH 9/1 (15 mL) was added to the crude extract and a solution of (trimethylsilyl)diazomethane (2M in heptane, 3.3 mL, 6.6 mmol, 1.25 eq) was added dropwise. The mixture was stirred 30 min and solvents were evaporated under reduced pressure. The crude of the reaction was purified under silica gel chromatography (95/5 to 80/20: pentane/EtOAc). 686 μg microgram of compound **13** was obtained (50% yield, 3 steps). *R_f_* = 0.57 (50/50: cyclohexane/EtOAc); ^1^H NMR (300 MHz, CDCl_3_): δ = 6.88 (dd, J = 10.7, 17.4 Hz, 1H), 5.71 (dd, J = 0.9, 17.4 Hz, 1H), 5.47 (d, J = 10.7 Hz, 1H), 3.62 (s, 3H), 2.64–2.61 (m, 2H), 2.40–2.37 (m, 2H), 2.24 (q, J = 7.5 Hz, 4H), 1.59–1.54 (m, 2H), 1.38–1.26 (m, 8H); ^13^C NMR (75 MHz, CDCl_3_): δ = 209.6, 174.2, 163.4, 141.7, 131.2, 120.7, 51.3, 34.0, 33.6, 29.3, 28.9 (2C), 28.7, 24.9, 24.8, 22.9; HRMS (ESI^+^) calculated for C_16_H_25_O_3_ [M+H]^+^ 265.1804, found 265.1819.

### (3S,6S,E)-oct-4-ene-3,6-diol (+)-14

At −5°C, a solution of BuLi (1.6 M in hexanes, 5.64 mL, 9 mmol, 1.3 eq) was added dropwise to a solution of freshly distillated 2,2,6,6-tetramethylpyperidine (1.52 mL, 9 mmol, 1.3 eq) in anhydrous *t*BuOMe (3.5 mL). The reaction was stirred 5 min at −5°C and 20 min at room temperature. After cooling at −5°C, (*S*)-1,2-epoxybutane (500 μL, 6.93 mmol, 1 eq) was added dropwise. The reaction was stirred 6 h at −5°C and overnight at room temperature. MeOH (10 mL) was added and then the reaction mixture was stirred 10 min and evaporated with celite® under vacuum. The crude was purified under silica gel chromatography (75/25 to 50/50: pentane/EtOAc) to give 149 mg of (+)-**14** (36% yield). *R_f_* = 0.1 (70/30: cyclohexane/EtOAc); ^1^H NMR (300 MHz, CDCl_3_): 5.61–5.59 (m, 2H), 4.00–3.94 (m, 2H), 2.35 (sl, 2H), 1.59–1.44 (m, 4H), 0.87 (t, J = 7.4 Hz, 3H); ^13^C NMR (75 MHz, CDCl_3_): δ = 133.8, 73.7, 30.0, 9.6; [α]^20^_*D*_ = +21.7 (c 10, CHCl_3_).

### (3R,6R,E)-oct-4-ene-3,6-diol (−)-14

Similar to the above, (*R*)-1,2-epoxybutane (500 μL, 6.93 mmol) gave access to compound (−)-**14** (160 mg, 38% yield). *R_f_* = 0.1 (70/30: cyclohexane/EtOAc); ^1^H NMR (300 MHz, CDCl_3_): 5.65–5.63 (m, 2H), 4.03–3.97 (m, 2H), 1.83 (sl, 2H), 1.60–1.47 (m, 4H), 0.89 (t, J = 7.4 Hz, 3H); ^13^C NMR (75 MHz, CDCl_3_): δ =133.7, 73.7, 30.0, 9.6; [α]^20^_*D*_ = −20.0 (c 10, CHCl_3_).

### 16-oxo-16-B_1_-PhytoP (16)

To a mixture of **13** (200 mg, 0.75 mmol, 1 eq) and pent-1-en-3-one (150 μL, 1.5 mmol, 2eq) in carefully degassed CH_2_Cl_2_ (15 mL) Hoveyda Grubbs 2nd generation catalyst (11.8 mg, 0.019 mmol, 0.025 eq) was added. The mixture was heated 4 h under reflux. To the reaction mixture celite® was added, followed by evaporation of the solvents without heating, and rapidly purified under silica gel chromatography (100/0 to 80/20: CH_2_Cl_2_/Et_2_O). The 16-oxo-16-B_1_-PhytoP methyl ester **16** was slightly degraded by silica gel and contaminated by byproducts. The mixture was used directly in the next step. To a solution of contaminated 16-oxo-16-B_1_-PhytoP methyl ester **16** in MeCN (8.5 mL) and phosphate buffer (8.5 mL, pH = 7) CALB (36 mg, 20% w/w) was added. The reaction mixture was stirred 5 h. After filtration, the crude mixture was extracted with EtOAc (3 × 50 mL). The organic layers were washed with brine (2 × 25 mL) and dried over anhydrous MgSO_4_. The solvents were removed under vacuum. The crude of the reaction was purified under silica gel chromatography (100/0 to 90/10: CH_2_Cl_2_/MeOH) to give 80.6 mg of 16-oxo-16-B_1_-PhytoP **17** (47% yield, 2 steps). *R_f_* = 0.39 (95/5: CH_2_Cl_2_/MeOH); ^1^H NMR (300 MHz, CDCl_3_): δ = 7.60 (d, J = 15.9 Hz, 1H), 6.53 (d, J = 15.9 Hz, 1H), 2.70–2.62 (m, 4H), 2.46–2.43 (m, 2H), 2.34–2.27 (m, 4H), 1.60–1.56 (m, 2 H), 1.40–1.23 (m, 8H), 1.13 (t, J = 7.2 Hz, 3H); ^13^C NMR (75 MHz, CDCl_3_): δ = 209.0, 200.5, 179.31, 160.1, 147.8, 134.0, 130.2, 34.7, 33.8, 33.7, 29.2, 28.8 (2C), 28.7, 25.3, 24.5, 23.3, 7.8; HRMS (ESI^+^) calculated for C_18_H_27_O_4_ [M+H]^+^ 307.1909, found 307.1895.

### 16-B_1_-PhytoP methyl ester (S)-15

To a mixture of **13** (90 mg, 0.34 mmol, 1 eq) and (-)-**14** (35 mg, 0.24 mmol, 0.7 eq) in carefully degassed CH_2_Cl_2_ (7 mL) Hoveyda Grubbs 2nd generation catalyst (5.3 mg, 0.0085 mmol, 0.025 eq) was added. The mixture was stirred for 4 days. New crops of catalyst (5.3 mg) were added each day. To the reaction mixture celite® was added, followed by evaporation of the solvents without heating, and rapid purification under silica gel chromatography (95/5 to 80/20: CH_2_Cl_2_/EtOAc). 53.6 mg of 16-B_1_-PhytoP methyl ester (*S*)-**15** was obtained (49% yield). *R_f_* = 0.26 (80/20: CH_2_Cl_2_/EtOAc); ^1^H NMR (300 MHz, CDCl_3_): δ = 6.74 (d, J = 15.7 Hz, 1H), 6.20 (dd, J = 5.8, 15.7 Hz, 1H), 4.23–4.18 (m, 1H), 3.59 (s, 3H), 2.60–2.57 (m, 2H), 2.39–2.33 (m, 3H), 2.21 (q, J = 7.5 Hz, 4H), 1.62–1.51 (m, 4H), 1.34–1.19 (m, 8H), 0.92 (t, J = 7.3 Hz, 3H); ^13^C NMR (75 MHz, CDCl_3_): δ = 209.5, 174.3, 163.2, 141.1, 140.1, 123.8, 73.4, 51.3, 33.9, 33.7, 30.0, 29.1, 28.9, 28.8, 28.6, 25.5, 24.7, 22.8, 9.5.

### *Ent*-16-B_1_-PhytoP methyl ester *(R)*-15

Similar to the above, from (+)-**14** the reaction gave *ent*-16-B_1_-PhytoP methyl ester (*R*)-**15** (52.6 mg, 53% yield). *R_f_* = 0.26 (80/20: CH_2_Cl_2_/EtOAc); ^1^H NMR (300 MHz, CDCl_3_): δ = 6.76 (d, J = 15.7 Hz, 1H), 6.21 (dd, J = 5.8, 15.7 Hz, 1H), 4.25–4.20 (m, 1H), 3.62 (s, 3H), 2.62–2.59 (m, 2H), 2.39–2.36 (m, 2H), 2.21 (q, J = 7.6 Hz, 4H), 2.13 (s, 1H), 1.64–1.53 (m, 4H), 1.37–1.23 (m, 8H), 0.95 (t, J = 7.3 Hz, 3H); ^13^C NMR (75 MHz, CDCl_3_): δ = 209.5, 174.3, 163.1, 141.2, 139.9, 123.9, 73.5, 51.3, 33.9, 33.7, 30.1, 29.1, 28.9, 28.8, 28.6, 25.5, 24.7, 22.8, 9.5.

### 16-B_1_-PhytoP (2)

To a solution of 16-B_1_-PhytoP methyl ester (*S*)-**15** (50 mg, 0.155 mmol, 1 eq) in MeCN (2.6 mL) and phosphate buffer (2.6 mL, pH = 7) CALB (1 mg, 2% w/w) was added. The reaction mixture was stirred for 1 day. After filtration, the crude was extracted with EtOAc (3 × 10 mL). The organic layers were washed brine (2 × 10 mL) and dried over anhydrous MgSO_4_. The solvents were removed under vacuum. The crude of the reaction was purified under silica gel chromatography (75/25 to 25/75: CH_2_Cl_2_/EtOAc) to give 42.4 mg of 16-B_1_-PhytoP**2** (88% yield). *R_f_* = 0.05 (50/50: CH_2_Cl_2_/EtOAc); ^1^H NMR (500 MHz, CDCl_3_): δ = 6.79 (d, J = 15.8 Hz, 1H), 6.25 (dd, J = 5.9, 15.8 Hz, 1H), 4.28 (q, J = 6.1 Hz, 1H), 2.65–2.63 (m, 2H), 2.43–2.41 (m, 2H), 2.32 (t, J = 7.3 Hz, 2H), 2.25 (t, J = 7.5 Hz, 2H), 1.65 (quint, J = 7.3 Hz, 2H), 1.61–1.58 (m, 2H), 1.36–1.33 (m, 2H), 1.33–1.24 (m, 6H), 0.97 (t, J = 7.4 Hz, 3H); ^13^C NMR (125 MHz, CDCl_3_): δ = 211.4, 180.2, 165.0, 142.7, 141.3, 125.4, 75.0, 35.3, 35.2, 31.4, 30.4, 30.0 (2C), 29.9, 27.0, 25.9, 24.2, 11.0; HRMS (ESI^+^) calculated for C_18_H_29_O_4_ [M+H]^+^ 309.2066, found 309.2064; [α]^20^_*D*_ = + 22.6 (c 10, CHCl_3_).

### *Ent*-16-B_1_-PhytoP (*ent*-2)

Similar to the above, from (*R*)-**15** the reaction gave *ent*-16-B_1_-PhytoP (*ent*-**2**) (36.8 mg, 77% yield). *R_f_* = 0.05 (50/50: CH_2_Cl_2_/EtOAc); ^1^H NMR (500 MHz, CDCl_3_): δ = 6.80 (d, J = 15.7 Hz, 1H), 6.25 (dd, J = 5.9, 15.7 Hz, 1H), 4.29 (q, J = 5.9 Hz, 1H), 2.67–2.63 (m, 2H), 2.43–2.41 (m, 2H), 2.32 (t, J = 6.9 Hz, 2H), 2.26 (t, J = 6.8 Hz, 2H), 1.68–1.56 (m, 4H), 1.41–1.24 (m, 8H), 0.98 (t, J = 7.2 Hz, 3H); ^13^C NMR (125 MHz, CDCl_3_): δ = 211.4, 180.1, 164.9, 142.7, 141.2, 125.4, 75.0, 35.3, 35.2, 31.4, 30.4, 30.03, 30.02, 29.9, 27.0, 25.9, 24.2, 11.0; HRMS (ESI^+^) calculated for C_18_H_29_O_4_ [M+H]^+^ 309.2066, found 309.2081; [α]^20^_*D*_ = − 22.1 (c 10, CHCl_3_).

## Result and discussion

**Scheme 2:**
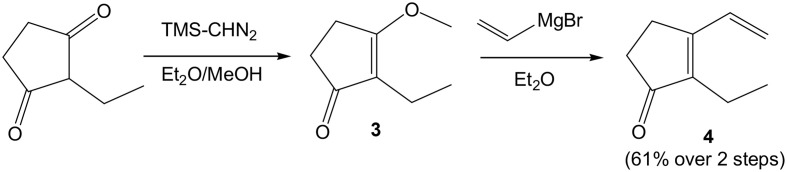
**Access to 2-ethyl-3-vinyl-cyclopentenone 4**.

**Scheme 3:**
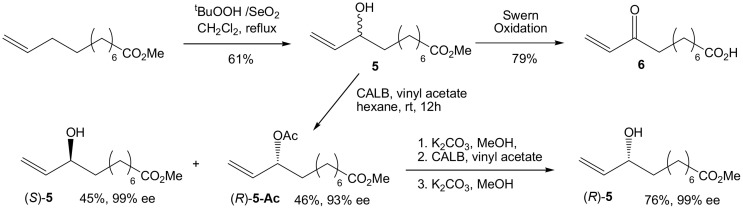
**Synthesis of alkene precursors**.

Our initial target was 9-L_1_-PhytoP, and while the ethyl group at the 2-position of 1,3-cyclopentanedione could be introduced using the Ramachary protocol (Ramachary and Kishor, 2008), we started with commercial and affordable 2-ethyl-1,3-cyclopentanedione (Scheme 2). Protection of one of the ketone groups was done by *O*-alkylation with commercial TMSCHN_2_ in a MeOH/Et_2_O solution to give the corresponding enol ether **3**. The latter is then treated with a solution of vinyl magnesium bromide in THF to provide the desired conjugated dienenone system **4** in 61% yield over 2 steps (Fisher et al., 1988). Having in hand **4**, the synthesis of racemic coupling partner **5** is easily achieved by allylic oxidation of commercial methyl 10-undecenoate with SeO_2_and *t*BuOOH in refluxing CH_2_Cl_2_ overnight in 61% yield (Scheme 3). The corresponding enone compound **6** was obtained by Swern oxidation sequence in 79% yield.

Enzymatic resolution of **5** was carried out using *Candida antartica* lipase B (CALB) with vinyl acetate in hexane on gram scale. After overnight conversion, non-reactive alcohol and acetate compounds are separated by silica gel to afford (*S*)-**5** in 99% ee and 45% yield and (*R*)-**5-Ac** in 46% yield and 93% ee. (*R*)-**5-Ac** can be submitted to a second enzymatic resolution, after methanolysis, CALB conversion and methanolysis to afford (*R*)-**5** in an improved 99% ee in 76% overall yield from (*R*)-**5-Ac**. Absolute configurations are determined using the corresponding mandelic ester derivatives (Chataigner et al., 1998).

Cross-metathesis (CM) between diene (or electron deficient diene) and alkene (or electron deficient alkene) are nowadays quite widespread in the literature (Grubbs, 2004), however CM between conjugated dienone and allylic alcohol, or even more complex electron deficient enone substrate have never been attempted to the best of our knowledge (Wojtkielewicz, 2013).

**Scheme 4:**
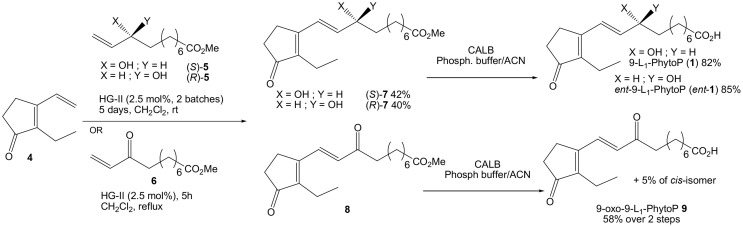
**Synthesis of 9-L_1_-PhytoP, its enantiomer and 9-oxo-L_1_-PhytoP**.

CM was initially investigated between allylic alcohol (*S*)-**5** and 2-ethyl-3-vinyl-cyclopentenone **4** (Scheme 4). Two second generation N-heterocyclic carbene ruthenium (II) complexes i.e., Grubbs 2nd generation (G-II) and Hoveyda-Grubbs 2nd generation (HG-II) were screened, and the best conditions were 2.5 mol% of catalyst in CH_2_Cl_2_ at room temperature for 2 days followed by another batch for 3 days. Later six other catalysts from Omega Cat System (M8_31_-SIPr, M8_32_-SIPr, M8_43_-SIPr, M7_3_-SIPr, M7_1_-SIPr, and M7_1_-SIMes) were tested, but HG-II proved to be the best giving 40% of our desired compound (*S)-**7***. G-II gave lower yield (33%) and the 6 others from Omega Cat System even lower (0–17%). Heating the reaction or microwave condition resulted in poor recovering yield. Homodimerized cyclopentenone of **4** were observed to a minor extent (10%). Similarly, CM of enantiomer (*R*)-**5** gave compound (*R*)-**7** in 42% yield. The final step in this short synthesis of 9-L_1_-PhytoP consisted of the enzymatic saponification of the carboxylic ester group of (*S*)-**7** using CALB (2% w/w) in MeCN/Phosphate buffer (pH = 7) (82% yield). *Ent*-9-L_1_-PhytoP was also recovered in 85% yield. Both syntheses permitted in a matter of 6–7 steps the recovery of more than 60 mg of those natural products.

To access 9-oxo-9-L_1_-PhytoP, which we believe is a potential metabolite of **1**, we decided to perform a CM between cyclopentenone **4** and enone **6**. Reaction conditions proved to be smoother than before as only one batch of 2.5 mol% of HG-II catalyst in refluxing CH_2_Cl_2_ for 5 h afforded the corresponding coupling product with complete consumption of starting materials. Purification was only performed to remove catalyst because substrate **8** slowly decomposed over silica gel. We also noted that dimers of **4** and **6** were formed in greater amount than from the previous CM with allylic alcohol **5**, even if we did not intend to recover them for further characterization. Saponification of 9-oxo-9-L_1_-PhytoP methyl ester **8** in MeCN/Phosphate buffer (pH = 7) with CALB provided after column chromatography 9-oxo-9-L_1_-PhytoP **9** (58% yield, 2 steps) contaminated with 5% of *Z* isomer.

**Scheme 5:**
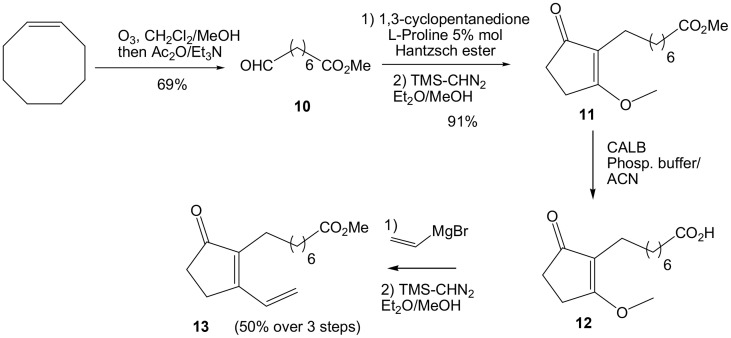
**Synthesis of cyclopentenone 13**.

To access our second target, 16-B_1_-PhytoP, the synthesis of methyl 8-oxooctanoate **10** was necessary to perform the reductive alkylation procedure to introduce the 2-alkyl-substituent of 1,3-cyclopentanedione (Scheme 5). Therefore, ozonolysis of cyclooctene followed by *in situ* transformation of the methoxy-hydroperoxide intermediate gave the desired aldehyde **10** in 68% yield on gram scale (Claus and Scheiber, 1986). The reductive alkylation of 1,3-cyclopentanedione with methyl 8-oxooctanoate **10** and Hantzsch ester under L-proline-catalysis (5 mol%) following Ramachary procedure (Ramachary and Kishor, 2008), provided 2-alkylated cyclopentane-1,3-dione at rt in CH_2_Cl_2_ for 4 h. Further reaction with TMSCHN_2_ in Et_2_O/MeOH furnished *O*-methylated compound **11** in 91% over 2 steps. In order to introduce the vinyl moiety, enzymatic saponification with CALB lipase was sought, leading to the carboxylic acid compound **12**, followed by vinyl magnesium bromide addition in THF. Late stage esterification with TMSCHN_2_ provided dienone compound **13** in 50% yield over 3 steps.

**Scheme 6:**
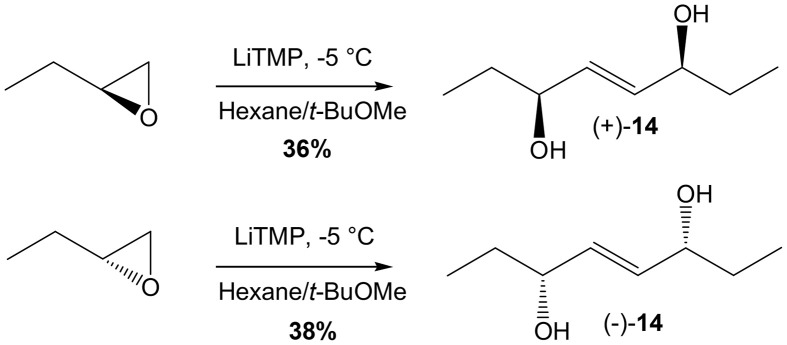
**LiTMP dimerization of 1,2-epoxybutane**.

**Scheme 7:**
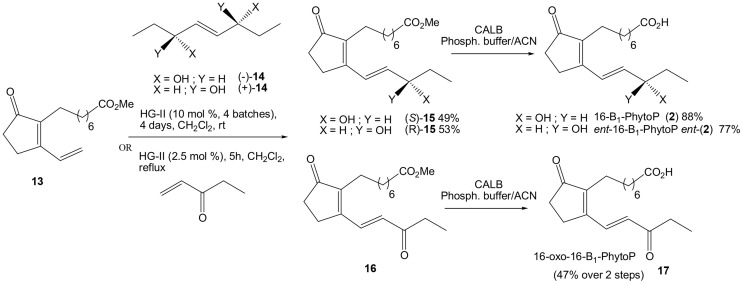
**Synthesis of 16-B_1_-PhytoP, its enantiomer and 16-oxo-B_1_-PhytoP**.

What remained to be accomplished was the metathesis CM between **13** and enantiomerically enriched penten-3-ol or corresponding penten-3-one. Resolution of penten-3-ol was already described in the literature by Sharpless asymmetric epoxidation (Moslin and Jamison, 2006), however we could not achieve the level of enantiopurity reported (66% vs. >90% ee). While no report for lipase enzymatic resolution of penten-3-ol was reported, we screened a panel of commercially available lipases but did not reach a significant level of enantiopurity either, which could certainly be explained due to the relatively small difference between the vinyl and the ethyl groups. However, it is well known that CM can also be performed starting with the homodimer of the olefin partner (Finnegan et al., 2006). Fortunately, a very simple procedure to access enantiomerically pure 2-ene-1,4-diols of such, from simple dimerization of terminal epoxides was reported by Hodgson's research group.(Hodgson et al., 2005) Therefore commercially available (*S*)-1,2-epoxybutane was added to LiTMP in *t*BuOMe at −5°C to give after purification homodimer (+)-**14** in 36% yield (Scheme 6). Similarly, (*R*)-1,2-epoxybutane yielded homodimer (-)-**14** in 38% yield. CM study started with cyclopentenone **13** (1 eq) and (−)-**14** (0.7 eq) in CH_2_Cl_2_ at rt for 4 days as previously described above, but with 2.5 mol% HG-II added every day (Scheme 7). These conditions afforded 16-B_1_-PhytoP methyl ester (*S*)-**15** in 49% yield. The last step consisted of enzymatic saponification with CALB yielding 16-B_1_-PhytoP **2** in 88% yield. The synthesis of *ent*-16-B_1_-PhytoP *ent*-**2** was achieved in similar conditions and yields from precursor (+)-**14**.

The last target, 16-oxo-16-B_1_-PhytoP **17** was obtained from commercially available pent-1-en-3-one (2 eq) and cyclopentenone **13** (1 eq) in refluxing CH_2_Cl_2_ for 4 h and only 2.5 mol% of HG-II to furnish 16-oxo-16-B_1_-PhytoP methyl ester **16**. CALB enzymatic saponification resulted in 16-oxo-16-B_1_-PhytoP **17** in 47% yield over 2 steps.

## Conclusion

In this paper, we have developed a rapid and flexible synthetic strategy for 2,3-substituted cyclopentenone phytoprostanes which can permit development of synthetic analogs from commercially available materials within a few steps. This strategy can compete with the Boland's one in terms of the number of steps and flexibility with a limitation in terms of yield in the CM compare to the Heck reaction (higher yield of coupling and scale), however no protecting group of the side chains was required in our strategy.

### Conflict of interest statement

The authors declare that the research was conducted in the absence of any commercial or financial relationships that could be construed as a potential conflict of interest.
